# Effects *of Musa sapientum* stem extract on experimental models of anxiety

**Published:** 2017

**Authors:** Aditya Jielella Reddy, Ashok Kumar Dubey, Shailendra Handu, Manocha Sachin, Pramod Kumari Mediratta, Qazi Ahmed Mushtaq

**Affiliations:** *Department of Pharmacology, SMS&R, Sharda University, Gr. Noida, NCR, India*

**Keywords:** Musa sapientum, Anxiety, Oxidative stress, Elevated plus maze, Mice

## Abstract

**Objective:**

The *Musa sapientum* (banana) plant extract has shown antioxidant activity in previous studies. Oxidative stress is one of the important factors implicated in the pathogenesis of anxiety disorders. The present study aimed to evaluate the anxiolytic activity of aqueous extract of *M. sapientum* stem (MSSE) in experimental models in mice.

**Material and Methods:**

Elevated Plus Maze method and locomotor monitoring by photoactometer were used. Animals were divided into five different groups (n=6/group). The vehicle, standard and the experimental groups were given distilled water (10 ml/kg), diazepam (1 mg /kg intraperitoneally) and incremental doses of 25, 50 and 100 mg/kg of MSSE, respectively, prior to the experiment. The standard group received diazepam.

**Results:**

The number of open arm entries and the duration of time spent in the open arms in the MSSE-treated groups increased significantly in a dose-dependent manner as compared to that of control group. The duration of time spent in closed arms in the MSSE-treated groups decreased significantly in a dose-dependent manner as compared to that of the control group. MSSE also decreased the locomotor activity significantly at all three test doses.

**Conclusion:**

The results of this study suggest an anxiolytic activity for MSSE, which make it a potential natural compound for treatment of anxiety disorders.

## Introduction

Anxiety disorders are the most common type of psychiatric problems with the highest lifetime prevalence of up to 28.8% and the earliest age of onset (11 years) among psychiatric disorders (Kessler, 2007 ▶). Pharmacotherapy of anxiety disorders often becomes challenging because the constant and disproportionate state of worry tends to be chronic in these disorders and is often recurring after remission (Byrne et al., 2008). Despite the availability of various groups of anti-anxiety drugs, their long-term use is generally hampered by dependence liability, tolerance, and cognitive and other behavioral side effects, apart from the economic burden of the chronic therapy (Cryan and Sweeney, 2011 ▶). Therefore, research on anti-anxiety potential of plant-derived compounds has been of particular interest, as an effective herbal therapy for anxiety may be a safer and less costly alternative for the patients with anxiety disorders, especially those who have a poor socioeconomic background.

Oxidative stress has been considered as one of the important factors implicated in the, still unclearly defined, pathogenesis of anxiety disorders. Several clinical and pre-clinical studies have shown the presence of higher levels of oxidative biomarkers and lower levels of antioxidant defense biomarkers in the brain and peripheral tissues in patients or models of anxiety as compared to the control groups (Emhan et al., 2015 ▶; Smaga et al., 2015 ▶).

*Musa sapientum*, commonly known as banana, is a herbaceous plant of Musaceae family. Different parts of the banana plant contain carotenoids, phenolic compounds, and biogenic amines like dopamine, serotonin, noradrenaline, tryptophan, and tyrosine, which are relevant to the pathophysiology of mental disorders (Pereira and Maraschin, 2015 ▶). Studies in experimental animal models have confirmed the therapeutic potential of the extracts obtained from different parts of this plant and these extracts have been shown to possess antioxidant activity (Adewoye et al., 2009 ▶; Tsai and Huang, 2016 ▶; Reddy et al., 2016 ▶). The antioxidant activity of *M. sapientum* reported by previous studies led us to assess of its potential anxiolytic activity. The present study was undertaken to evaluate the anxiolytic activity of *M. sapientum* stem extract (MSSE) in experimental models in mice, which, to the best of our knowledge, has not been done yet.

## Materials and Methods

**Animals**

Swiss albino mice of either sex weighing between 25-30g were used in this study after obtaining the permission from the Institutional Animal Ethics Committee. The animals were housed in the central animal house of the institution, under standard laboratory conditions (12hr:12hr light:dark cycle, temperature 22±2°C and 30-70% humidity) and had free access to pellet diet and water, *ad libitum*. Prior to the day of experiment, animals were kept on fast overnight, though water was given *ad libitum*. The animals were acclimatized to laboratory conditions prior to experimentation and proper care was taken of the animals as per the standard guidelines of Committee for the Purpose of Control and Supervision of Experiments on Animals (CPCSEA). 

**Plant material and preparation of aqueous extract**

Fresh banana stems were collected locally in the month of October, 2015 and the stem sample was authenticated by an authorized center (National Institute of Science Communication and Information Resources). The central pale white stem under the concentric layers of leaf sheaths was cut into small pieces. Aqueous extract was prepared from these parts, lyophilized and stored in refrigerator at 4˚C, to be used in the experiments.

**Animal models for anxiety**

*Elevated plus maze (EPM)*

The EPM apparatus consisted of two open arms (30×10 cm each), two enclosed arms (30×10×20 cm each) and a central platform (10 × 10 cm), arranged in such a way that the two arms of each type were opposite to each other forming a plus sign. The maze was elevated 40 cm above the floor. Forty five min after the administration of test dose, each animal was placed in the center of the maze facing one of the enclosed arms and allowed to freely move into any of the arms. During the 5-min test period, the number of open and enclosed arms entries was recorded. The time spent in open and enclosed arms was also recorded. Entry into an arm was considered when the animal placed all four paws on the arm. Increase in the open arms entries is suggestive of an anxiolytic effect (Pellow and File, 1986 ▶; Barrett, 1991 ▶).

*Open Field Test*

The locomotor activity was measured using a digital actophotometer (30 × 30 × 25 cm). The apparatus consisted of a cage, with beams of light falling on the corresponding photoelectric cells. When an animal is placed in the cage, its movement interrupts the light beams and each interruption is displayed digitally. The number of interruptions for each animal on test dose was counted for ten min and was considered as a measure of locomotor activity of the animal. A decrease in spontaneous motor activity is suggestive of sedative-hypnotic effect due to CNS depressant action of a drug. 

Both of the above-mentioned tests for anxiety were carried out in five different groups (n=6/group) of mice. Based on the previous reports and pilot studies, three test groups were orally administered with incremental doses of 25, 50 and 100 mg/kg of MSSE, respectively, while the vehicle and the standard drug group were administered with distilled water (10 ml/kg) and diazepam (1mg/kg, intraperitoneally), respectively, 45 min prior to the experiments (Dikshit et al., 2011 ▶).

**Statistical analysis**

Data were presented as mean ± S.E.M (standard error of mean) and analyzed by one-way analysis of variance (ANOVA) followed by *post-hoc* Tukey's test. Statistical analysis was performed using SPSS version 17. Differences with p values <0.05 were considered significant. 

## Results

**Effect of MSSE on number of closed arms entries in EPM method in mice**

The number of enclosed arm entries in control group decreased by MSSE 25 and 50 mg/kg and increased by MSSE 100 mg/kg in the treated groups. There was an increase in the number of entries to the diazepam-treated group. The results of enclosed arm entries were inconsistent and there was no significant change in the number of entries in any group as compared to those in the control group. The results are presented in Table 1. 

**Table 1:** Effect of MSSE on the number of enclosed arms entries by EPM method in mice

**Group**	**Treatment**	**No. of closed arm entries** **(Mean±SEM)**
Control (distilled water)	10 ml/kg, po	4.67 ± 0.34
MSSE	25 mg/kg, po	4.34 ± 0.21
MSSE	50mg/kg, po	4.00 ± 0.63
MSSE	100mg/kg, po	5.00 ± 0.37
Diazepam	1mg/kg, ip	7.00 ± 0.37*

* p<0.05 as compared to control group

**Effect of MSSE on number of open arms entries in EPM method in mice**

MSSE increased the number of open arm entries by the animals in a dose-dependent manner. The results are presented in Table 2. The number of open arm entries was statistically significant (p<0.05) increased following treatment with the highest dose of MSSE (100 mg/kg) as compared to the control group. Diazepam produced a significant (p<0.01) increase in the number of open arm entries when compared to the control group. Diazepam more markedly increased the number of open arm entries as compared to the highest dose of MSSE (100 mg/kg) and the difference was statistically significant (p<0.05).

**Table 2:** Effect of MSSE on the number of open arms entries by EPM method in mice

**Group**	**Treatment**	**No. of open arm entries** **(Mean±SEM)**
Control (distilled water)	10 ml/kg , po	2.34 ± 0.21
MSSE	25 mg/kg, po	2.50 ± 0.22
MSSE	50mg/kg, po	3.00 ± 0.45
MSSE	100mg/kg, po	4.34 ± 0.56*
Diazepam	1mg/kg, ip	7.17 ± 0.48**

* p<0.05 and

** p<0.01 compared to control group, respectively**.**

**Effect of MSSE on time spent in enclosed arms in EPM method**

MSSE produced a dose-dependent decrease in the duration of time spent by the animals in enclosed arm. The results are presented in Table 3. The difference in the duration of time spent in enclosed arms was significant (p<0.01) when comparing middle and higher dose of MSSE (50 and 100 mg/kg) with the control group. Diazepam significantly (p<0.01) decreased the duration of time spent in enclosed arms as compared to the control. Diazepam more markedly decreased the duration of time spent in enclosed arms as compared to the higher dose of MSSE (100 mg/kg) and the difference was statistically significant (p<0.05). 

**Table 3:** Effect of MSSE on the time spent in enclosed arms by EPM method

**Group**	**Treatment**	**Time spent in enclosed arm (sec) (Mean±SEM)**
Control (distilled water)	10 ml/kg, po	200.33 ± 3.29
MSSE	25 mg/kg, po	191.33 ± 2.85
MSSE	50mg/kg, po	176.83 ± 2.05**
MSSE	100mg/kg, po	171.17 ± 4.09**
Diazepam	1mg/kg, ip	148.00 ± 2.13**

** p<0.01 as compared to control group.

**Effect of MSSE on time spent in open arms in EPM method**

The results are presented in Table 4. MSSE produced a dose-dependent increase in the duration of time spent in open arms. The duration of time spent in the open arms was significantly different following treatment with MSSE 50 and 100 mg/kg (p<0.05 and p<0.01 respectively), when compared with the control group. Diazepam significantly increased the duration of time spent in open arms (p<0.01) as compared to the control. The increase in the time spent in the open arms induced by diazepam was more as compared to the highest dose of MSSE (100 mg/kg) and the difference was statistically significant (p<0.05). 

**Table 4:** Effect of MSSE on the time spent in open arms assessed by EPM method

**Group**	**Treatment**	**Time spent in open arm (sec)** **(Mean±SEM)**
Control (distilled water)	10 ml/kg, po	70.84 ± 3.94
MSSE	25 mg/kg, po	75.17 ± 3.76
MSSE	50mg/kg, po	92.33 ± 3.27*
MSSE	100mg/kg, po	101.67 ± 2.00**
Diazepam	1mg/kg, ip	133.00 ± 3.52**

* p<0.05 and

** p<0.01 compared to control group, respectively.

**Table 5:** Effect of MSSE on the spontaneous locomotor activity in mice

**Group **	**Treatment **	**No. of photo beam interruptions ** **(Mean±SEM) **
Control (distilled water)	10 ml/kg, po	508.17 ± 7.25
MSSE	25 mg/kg, po	448.83 ± 8.06**
MSSE	50mg/kg, po	378.17 ± 10.24**
MSSE	100mg/kg,po	178.17 ± 5.86**
Diazepam	1mg/kg, ip	154.00 ± 9.88**

** p<0.01as compared to control group.

**Effect of MSSE on locomotor activity**

MSSE decreased the number of photo beam interruptions in a dose-dependent manner (Table 5). The number of photo beam interruptions induced by all doses of MSSE (i.e. 25, 50, and 100 mg/kg) was significantly different (p<0.01) from that of the control group. Diazepam decreased the number of photo beam interruptions significantly (p<0.01) when compared to control. Although diazepam was more potent in decreasing the number of photo beam interruptions as compared to the highest dose of MSSE (100 mg/kg), the difference was not statistically significant (p>0.05).

## Discussion

Pharmacotherapy that is currently available for anxiety disorders has its limitations due to various adverse effects including the risk of developing dependence and tolerance after prolonged use. The chronic therapy of this condition, which tends to recur and is often persistent, also has negative economical implications. This has raised interest for research in the area of herbal psychopharmacology. Polyphenolic compounds are known to have influence on various aspects of mental health including brain plasticity, behavior, mood, and cognition (Trebaticka and Durackova, 2015 ▶). It has been shown in the earlier studies that phytochemicals from medicinal plants help to maintain normal physiological function of the major inhibitory neurotransmitters (Kumar and Khanum, 2012 ▶). 

*Musa sapientum*, the banana plant commonly available in South East Asia has been shown to possess antioxidant properties in a few previous studies. A decrease in lipid peroxidation and an increase in serum superoxide dismutase could be seen in the experimental models of diabetes treated with the plant extract, indicating an antioxidant activity for the plant’s extract (Adewoye et al., 2009 ▶). In another study, the plant extract was found to raise the glutathione and superoxide dismutase level while preventing increase in malondialdehyde, a lipid peroxidation marker and it was also shown to be hepatoprotective due to its antioxidant property (Dikshit et al., 2011 ▶). One study demonstrated the plant extract to possess antiulcerogenic activity and attributed the trait to its antioxidant activity (Goel et al., 2001 ▶).

Clinical and preclinical evidence indicate that mood disorders like anxiety and depression are characterized by higher levels of oxidative biomarkers and lower levels of antioxidant defense biomarkers in the brain and peripheral tissues (Emhan et al., 2015 ▶). Oxidative stress has often been cited as one of the important factors involved in the genesis of anxiety disorders. Patients with anxiety disorders have been found to have significantly higher total oxidant status and oxidative stress index as compared to the control groups (Emhan et al., 2015 ▶; Sajja et al., 2015 ▶).

Evaluation of antioxidative status, by measuring the redox potential of urine, has shown that normal anxiety state values corresponded to low urine redox potentials, whereas higher anxiety states are associated with high urinary redox potential (Grases et al., 2014 ▶). Studies using knockout or over-expression of antioxidant enzymes have found oxidative stress to be directly involved in the pathogenesis of anxiety-like behavior, with the related factors of oxidative stress such as impaired function of different mitochondrial proteins, inflammatory cytokines, and neurotrophic factors also playing important roles in the disorder, suggesting that a therapy specifically aimed at reducing the reactive species production may have a beneficial effect in reducing anxiety (Krolow et al., 2014 ▶).

EPM is commonly used for screening anxiety-modulating drugs. The test is based on the natural tendency of the rodents to avoid open spaces and uses the conflict between exploration and avoidance. Provoked behavior in the EPM has been suggested to include elements of fear, exploration, and approach / avoidance conflict reflecting anxiety and motor activity. Suppression of the tendency of the mice to prefer the closed arms is indicative of anxiolytic activity of the test compound (Michel, 2015 ▶). 

This study was undertaken to evaluate the potential of MSSE as an anxiolytic compound considering its already reported antioxidant activity. The results of the study suggest significant anxiolytic activity for MSSE as compared to the control group at the dose range of 50-100 mg/kg. As expected, diazepam, a benzodiazepine drug, produced significant anxiolytic effect as compared to the control group, consistent with the results of numerous earlier studies, which have established diazepam’s anxiolytic effects in various screening procedures, including the EPM method (Hajhashemi et al., 2010 ▶). MSSE also significantly decreased the spontaneous motor activity, assessed by the actophotometer, which was comparable to the effect of diazepam. Though the effects on locomotor activity were comparable to the effects of diazepam, the anxiolytic effect assessed by EPM was significantly less than that of diazepam. It is not possible to draw inference from these results to the mechanistic resemblance of MSSE with benzodiazepines. 

This was a preliminary exploratory study, carried out to evaluate the anxiolytic potential of MSSE, based on the background of previous studies showing its antioxidant properties. The antioxidant activity may have played a role in the significant anxiolytic effects produced by MSSE in the present study but there could be other independent or interlinked mechanisms involved which need to be further elucidated in future studies.

Results from this experimental study show the presence of a significant dose-dependent anxiolytic activity for MSSE, suggesting it to be a potential natural compound for treatment of anxiety disorders. However, further studies are required to identify the active constituents of the extract and ascertain its effectiveness and mechanism of action in anxiety.
